# Predictors of subclinical atherosclerosis in HIV

**DOI:** 10.1186/s12879-022-07976-1

**Published:** 2023-01-10

**Authors:** Julia Fernández Soto, Manuel J. Romero-Jiménez, José Carlos Alarcón García, Elena Bonet Estruch, José Luís Sánchez Ramos, Miguel Ángel Castaño López

**Affiliations:** 1 Internal Medicine Service, Lipid and Vascular Risk Unit, Infanta Elena Hospital, Doctor Pedro Naranjo S/N Street, 21007 Huelva, Spain; 2 Infectious Diseases Unit, Infanta Elena Hospital, Huelva, Spain; 3 Clinical Analysis Unit of the Infanta Elena Hospital, Huelva, Spain; 4 Nursing Department, University of Huelva, Huelva, Spain

**Keywords:** HIV, CD4/CD8 ratio, LDL-cholesterol, Tobacco, Subclinical atherosclerosis, Vascular mortality

## Abstract

**Background:**

Cardiovascular disease is a major cause of morbidity and mortality in people with HIV. The detection of subclinical atherosclerosis through vascular ultrasound allows us to identify patients at an increased risk of cardiovascular disease as a primary prevention strategy; this test is not routine. Our objective is to identify predictors of subclinical atherosclerosis in a population with HIV.

**Methods:**

People with HIV infection were selected for primary prevention and underwent carotid and femoral ultrasound to detect atheromatous plaques. Logistic regression analysis including vascular risk factors was performed to predict the presence of atherosclerosis.

**Results:**

One hundred eighty-three patients were included, 54% of whom were smokers; the mean duration of HIV infection was 9.52 years, and all patients were undergoing antiretroviral treatment. Subclinical atherosclerosis was present in 62.29% of the patients; 83.32% had plaque in the carotid territory, 57.93% in the femoral territory and 25.6% in both vascular territories. Compared to those without atherosclerosis, patients with atherosclerosis were on average 5.35 years older (53.86 vs. 48.51, p < 0.001) and had a higher prevalence of smoking (63.23% vs. 39.12%, p = 0.020) and a CD4/CD8 ratio below 0.7 (44.23% vs. 29.02%, p = 0.043). A CD4/CD8 ratio lower than 0.3 was always associated with subclinical atherosclerosis (95% confidence interval (CI): 83.9–100%). The inclusion of smoking, the CD4/CD8 ratio and age in the logistic regression analysis led to a diagnostic yield of 72% measured by the area under the receiving operator characteristic (ROC) curve (95% CI: 64–80%).

**Conclusions:**

Tobacco use, age and a CD4/CD8 ratio below 0.7 allow prediction of the presence of subclinical atherosclerosis in primary prevention. A CD4/CD8 ratio below 0.3 was a diagnostic indicator of atherosclerosis in HIV patients undergoing primary prevention in our sample.

## Background

The development of antiretroviral therapies (ARTs) and their greater accessibility have allowed better control of human immunodeficiency virus (HIV) infection and diseases related to acquired immunodeficiency syndrome (AIDS). The life expectancy of people with HIV has therefore increased and is currently similar to that of the general population [1]. Consequently, cardiovascular disease (CVD) is currently one of the main causes of morbidity and mortality [2] in this group of people.

People with HIV have an increased vascular risk, which extends beyond what can be explained by recognized traditional risk factors [3] and may be related to HIV infection itself and to antiretroviral treatment.

Despite effective antiretroviral treatment, HIV infection results in degrees of residual viremia and is associated with persistent immune activation, which generates a direct chronic inflammatory effect on artery walls. Along with other related mechanisms, such as coagulation failure, dyslipidaemia, alteration of the elasticity of arterial walls and endothelial dysfunction, residual viremia accelerates the formation of atheromatous plaques [4, 5], which are precursors of the onset of cardiovascular events.

Atheromatosis detection in the carotid and femoral territories by ultrasound in people with HIV without a history of CVD, which is called subclinical atheromatosis (SAT), would have value in the prediction of new cardiovascular events [6]. However, despite being an innocuous and low-cost technique, it is not always accessible to clinicians. Therefore, the identification of participants with a high probability of presenting with SAT would be of interest to select those with a greater risk of suffering CVD in the future.

The purpose of our study was to identify predictive factors of SAT in people with HIV without a history of established CVD.

## Methods

### Study design and population

A cross-sectional and multicentre study was carried out by the Lipid and Vascular Risk Unit of the Infanta Elena Hospital together with the Infectious Diseases Units of the Infanta Elena and Juan Ramón Jiménez Hospital in Huelva, Spain. This study was approved by the Research Ethics Committee of the province of Huelva with the code JFS-ATE-2017-01.

The HIV population of the province of Huelva is registered in a database, which currently includes a total of 1208 patients and is managed by the computer program Hospital Control Application (Aplicación de Control Hospitalario—AC&H™) used by the Infectious Diseases Units of the Infanta Elena and Juan Ramón Jiménez Hospitals in Huelva. This program is widely used in several hospitals of the National Health System, is a component of the VIH-Aplicación de Control Hospitalario (VACH) cohort [7] and collects demographic and clinical data, HIV transmission category, serological and immunovirological data, history of antiretroviral treatments performed, comorbidities, opportunistic diseases and specific data on diseases unrelated to HIV.

To enter the study, participants had to meet the following inclusion criteria: HIV infection with more than 10 years of evolution, age between 18 and 65 years and signed informed consent.

The presence of any of the following criteria excluded the participants from the study: class C cirrhosis according to the Child‒Pugh classification; positive serology for hepatitis B virus (HBV); type 1 diabetes mellitus; renal failure (glomerular filtrate < 60 mL/min by Chronic Kidney Disease Epidemiology Collaboration); recorded history of vascular disease including acute coronary syndrome, acute myocardial infarction, aortic aneurysm, ischaemic or haemorrhagic stroke, transient ischaemic attack (TIA), peripheral arterial disease (intermittent claudication, pain or paraesthesia at rest, trophic lesions, established gangrene), coronary revascularization (percutaneous or bypass) or any other arterial revascularization procedure, plaque visualized on coronary angiography or carotid ultrasound or arterial stenosis at any level (> 50%).

After informed consent was signed by each patient, an imaging study of the carotid (common carotid artery, bulbar, right and left internal and external carotid arteries) and femoral (common and superficial vessels, also on both sides) vascular territories was performed with a Toshiba Medical Systems Aplio XG scanner. The presence of atherosclerotic plaque was assessed by high-resolution colour vascular Doppler ultrasound following the Mannheim consensus protocol of 2004–2006 [8], which defines an atheroma plaque as a focal structure that encroaches on the arterial lumen by at least 0.5 mm.

The measurements obtained from the digital images were performed by the same experienced technician who did not know the clinical characteristics of the participants.

The results were included in a database created for this purpose, which complies with confidentiality guarantees and data protection regulations. Access to the database by professionals is restricted, and the use of data for this research was carried out anonymously.

We evaluated the presence of the classic risk factors associated with atherosclerosis in each participant: age, arterial hypertension, diabetes, dyslipidaemia, active smoking and a family history of early cardiovascular events following the definition criteria of the European Atherosclerosis Society [9]. We also considered analytical parameters such as total cholesterol, cholesterol bound to low- (LDL-C) and high-density lipoproteins (HDL-C), triglycerides, CD4/CD8 ratio at the beginning of treatment, CD4 nadir and viral suppression time, which was defined as < 200 copies/mL or < 50 copies/mL of HIV-1 RNA in plasma according to the method used (< 200 copies/mL from 1996 to 2006 and < 50 copies/mL subsequently), as well as the history of coinfection by hepatitis C virus (HCV), immunoglobulin G (IgG) positive for cytomegalovirus (CMV) and ART history. We considered treatments performed when any of the following therapeutic groups were used for at least one year: non-nucleoside reverse transcriptase inhibitors (NNRTIs), nucleoside and nucleotide reverse transcriptase inhibitors (NRTIs), protease inhibitors (PIs) and integrase strand transfer inhibitors (INSTIs).

### Statistical analysis

Descriptive statistics were performed for all variables: the mean and standard deviation for continuous variables and frequencies with percentages for categorical and ordinal variables.

Student t tests were used to compare parametric quantitative variables, and Mann‒Whitney U tests were used for variables that did not follow normality. The chi-square test (or Yates’ correction for continuity) was used for categorical and ordinal variables.

Through logistic regression analysis, we evaluated possible independent predictors of the presence of atheromatous plaque among all the variables studied using the Wald test. The diagnostic performance of the model was obtained by the area under the receiver operating characteristics (ROC) curve (AUC).

The cutoff points were established using the ROC curve. A cutoff point of 0.3 was chosen to achieve a specificity of 100%, and a cutoff point of 1.7 was chosen to achieve a sensitivity of 0%.

The cutoff point 0.7 was selected because it contributed the most information to the model and yielded the highest diagnostic performance. Cutoff points 0.3 and 1.7 did not contribute additional information.

All analyses were performed using the statistical package R Commander UCA, version 4.4.1, released September 26, 2021, from the University of Cádiz (Spain). An alpha error of 0.05 was considered statistically significant.

## Results

Of the 1208 HIV patients registered in the AC&H database, records for 809 were discarded because they met the following exclusion criteria: 38 were older than 65 years or younger than 18 years, 427 had HIV infection with less than 10 years of evolution, 54 had suffered previous CVD, 147 had renal failure (filtering less than 60 mL/min), and 143 had HBV infection.

**Fig. 1:**
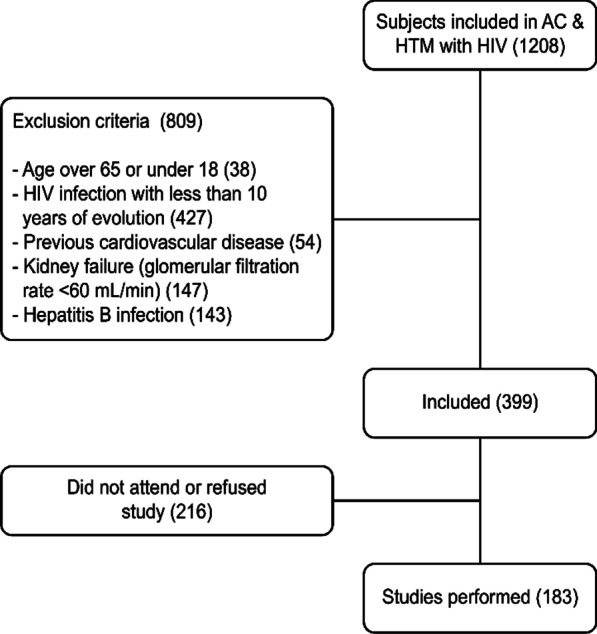
Flowchart of the study: participant selection with the inclusion and exclusion criteria. AC&H™: Hospital Control Application (Aplicación de Control Hospitalario) used by the Infectious Disease Units of the hospitals of the province of Huelva

A total of 399 patients were selected, 183 of whom agreed to participate in the study and signed the informed consent form (Fig. 1).

We included data from 183 patients collected from October 2020 to July 2021. A total of 114/183 (62.29%) presented with SAT. A total of 95/114 (83.32%) had plaque in the carotid territory, and 66/114 (57.93%) had plaque in the femoral territory. A total of 47/114 (41.23%) had plaques in both vascular territories.

The general characteristics of the HIV population with and without SAT are described in Table 1. We found differences between the two groups in age (53.86 vs. 48.51, p < 0.001) and smoking behaviour (63.23% vs. 39.12%, p = 0.020). Other vascular risk factors, such as female sex, diabetes, dyslipidaemia, arterial hypertension and family history of early CVD, were more frequent in the SAT group, although without statistical significance. Lipid profiles were similar in both groups (Table 1).

**Table 1:** General characteristics of the HIV population: distribution according to the presence atheromatous plaque

Characteristics		Subjects without atherosclerotic plaque N = 69 (37.70%) Mean; SD N (%)	Subjects with atherosclerotic plaque N = 114 (62.29%) Mean; SD N (%)	p value
Age (years)		48.51; 7.96	53.86; 8.20	< 0.001
*Sex*				
Female		20 (35.1%)	37 (64.9%)	0.623
Male		49 (38.9%)	77 (61.1%)	
*Hypertension*				
Yes		21 (31.8%)	45 (68.2%)	0.217
No		48 (41%)	69 (59%)	
*Type 2 diabetes mellitus*				
Yes		9 (29%)	22 (71%)	0.274
No		60 (39.5%)	92 (60.5%)	
*Dyslipidaemia*				
Yes		22 (29.3%)	53 (70.7%)	0.052
No		47 (43.5%)	61 (56.5%)	
*Active smoking*				
Yes		27 (27.3%)	72 (72.7%)	0.002
No		42 (50%)	42 (50%)	
*Family history of early CVD*				
Yes		2 (18.2%)	9 (81.8%)	0.168
No		67 (39%)	105 (61%)	
Total cholesterol (mg/dL)		212.58; 46.87	218.81; 48.17	0.393
LDL-C (mg/dL)		145.50; 43.08	149.61; 46.30	0.551
HDL-C (mg/dL)		52.88; 16.13	54.91; 15.66	0.402
Triglycerides (mg/dL)		154.14; 121.31	145.24; 90.44	0.572
Years of HIV infection**		18.04; 7.98	22.11; 8.06	0.001
Duration of viral suppression* (years)		9.01; 4.76	10.03; 4.29	0.161
CD4 cell nadir (cells/µL)		231.94; 175.85	166.17; 141.4	0.010
*CD4/CD8 < 0.7*				
Yes		13 (22%)	46 (78%)	0.003
No		56 (45.2%)	68 (54.8%)	
*CD4/CD8 < 0.3*				
Yes		0 (0%)	17 (100%)	0.001
No		69 (41.6%)	97 (58.4%)	
*CD4/CD8 > 1.7*				
Yes		18 (100%)	0 (0%)	< 0.001
No		51 (30.9%)	114 (69.1%)	
*Positive anti-HCV IgG*				
Yes		17 (26.2%)	48 (73.8%)	0.017
No		52 (44.1%)	66 (55.9%)	
*IgG anti-CMV*				
Yes		38 (36.5%)	66 (63.4%)	0.288
No		31 (39.2%)	48 (60.7%)	
*Treatment regimen with PI*				
Yes		30 (29.4%)	72 (70.6%)	0.009
No		39 (48.1%)	42 (51.9%)	
*Treatment regimen with NRTI*				
Yes		29 (43.3%)	34 (30.6%)	0.087
No		40 (33.3%)	80 (66.6%	
*Treatment regimen with NNRTI*				
Yes		38 (56.71%)	54 (48.60%)	0.297
No		31(34.06%)	60 (65.93%)	
*Treatment regimen with INSTI*				
Yes		27 (44.2%)	34 (55.74%)	0.442
No		42 (34.4%)	80 (65.5%)	

SD, standard deviation; CVD, cardiovascular disease; LDL-C, low-density lipoprotein cholesterol; HDL-C, high-density lipoprotein cholesterol; HCV, hepatitis C virus; IgG, immunoglobulin G; CMV, cytomegalovirus; NNRTI, non-nucleoside reverse transcriptase inhibitor; NRTI, nucleoside reverse transcriptase inhibitor; PI, protease inhibitor; INSTI, integrase strand transfer inhibitor

(*) Viral suppression time, defined as < 200 copies/mL or < 50 copies/mL of HIV-1 RNA in plasma, according to the method used (< 200 copies/mL from 1996 to 2006 and < 50 copies/mL later)

(**) Until the time a vascular ultrasound study was conducted

In terms of characteristics related to HIV infection, the years of exposure to HIV for patients with SAT compared to those without SAT were 22.28 vs. 18.42 (p = 0.002). A low CD4 nadir was associated with the presence of SAT compared to the absence of SAT (166.17 vs. 231.94 cells/µL, p = 0.013).

We found an inverse relationship between the CD4/CD8 ratio and SAT; a CD4/CD8 ratio lower than 0.7 was associated the presence of SAT compared to the absence of SAT (44.23% vs. 29.02%, p = 0.043). All patients with a CD4/CD8 ratio < 0.3 had SAT (95% confidence interval (CI): 83.9–100%), and none of the patients with a CD4/CD8 ratio > 1.7 showed SAT (95% CI: 84.7–100%).

The history of HCV coinfection was higher in the SAT group (42.12% vs. 24.64%, p = 0.017). Regarding the treatments performed, we found that PI use was higher in the SAT group (63.21% vs. 41.83%, p = 0.006). The use of reverse transcriptase (RT) inhibitors, both analogues and non analogues, was more frequent in the group without SAT, although without statistical significance. The use of integrase inhibitors was more frequent in the SAT group, but the difference was not significant. The duration of viral suppression measured in years was similar in both groups (10.03 vs. 9.01, p = 0.161). No differences in positive CMV serology were found.

Adjusting for all the variables present in the model, those that provided the most predictive information were selected: age, smoking and CD4/CD8 < 0.7. PI use and the rest of the variables explored were not included in the model.

**Fig. 2:**
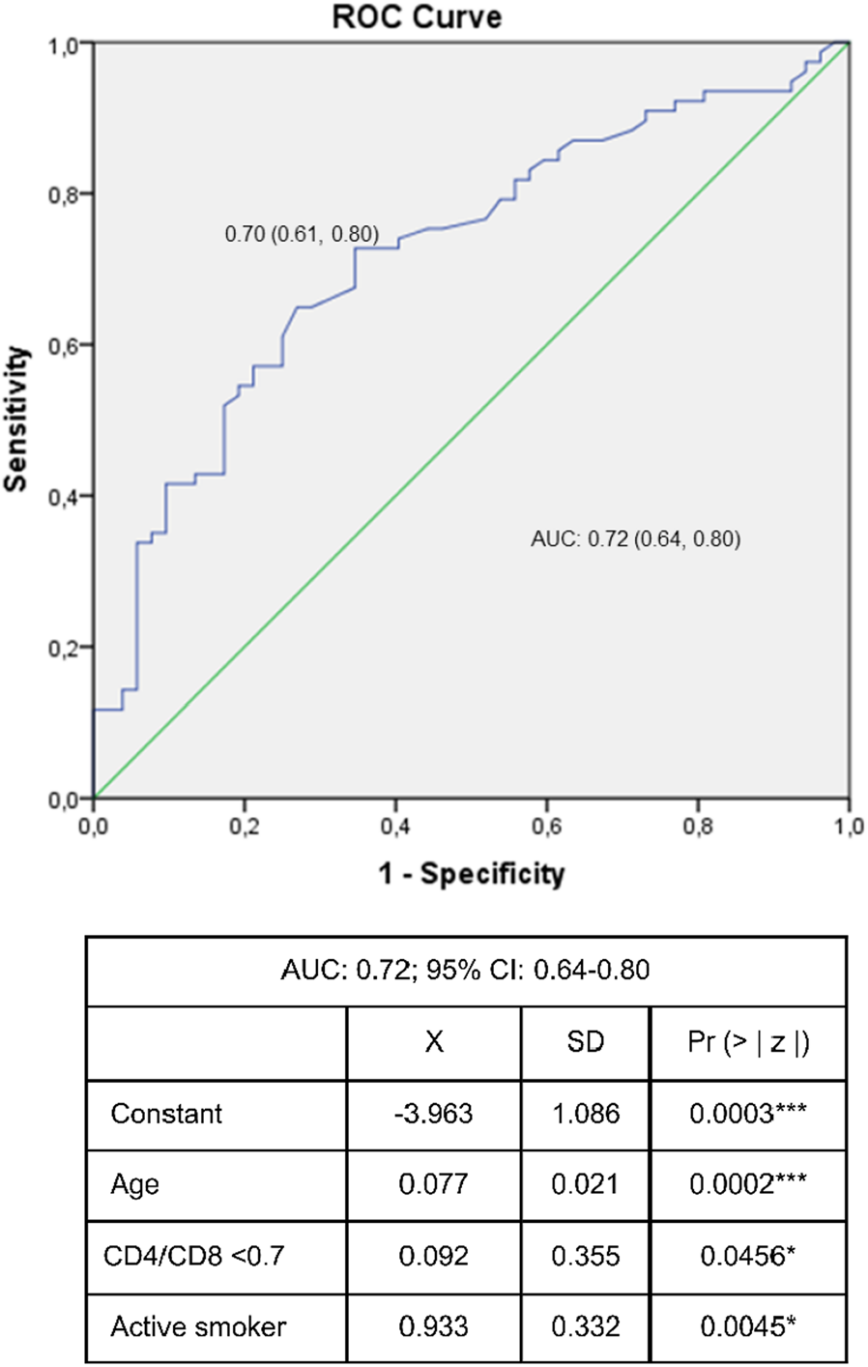
ROC curve for subclinical atheromatosis (SAT) detection in HIV participants without previous cardiovascular disease. Receiver operating characteristics curve evaluating the accuracy SAT detection in participants with HIV without previous CVD. AUC: area under the curve (95% confidence interval); X: mean; SD: standard deviation. The probability of presenting atheromatous plaque was calculated using the following formula: $$\mathrm{P }(\mathrm{Plaque}) =\frac{1}{{1+e}^{-(3.963-0.077*Age-0.092*CD4/CD8\left(if<0.7\right)-0.933*(if active smoker)}}$$ where P: the probability of presenting atheromatous plaque; e: exponential constant; Age: age in years; CD4/CD8: 1 if CD4/CD8 < 0.7; Active smoker: 1 if an active smoker

Including active smoking, age and a CD4/CD8 ratio < 0.7 in a prediction equation for subclinical atherosclerosis by means of logistic regression achieved good predictive power. As shown in Fig. 2, the diagnostic performance of this prediction by means of the AUC was 72% (95% CI: 64–80%).

## Discussion

The increase in the incidence of CVD in people with HIV and underestimation of the CVD risk through commonly used tools necessitate exploration of new resources to adequately assess vascular risk in these patients. SAT is the pathophysiological basis of CVD. The high prevalence of SAT among the HIV population may explain the greater number of cardiovascular events in people with HIV [10].

In our cohort, the prevalence of SAT among HIV participants without previous CVD was 62.29%, which is much higher than that published in the literature. In a recent meta-analysis by Liu et al. [11], a prevalence of carotid SAT between 31.6% and 37% was reported.

The higher frequency of atheromatous plaque recorded in our study can be explained by several factors.

### Factors related to the population studied

Our study was developed in Europe, where the prevalence of traditional cardiovascular risk factors (CVRFs), such as smoking, obesity and alcohol abuse, is high [12]. Most of the participants in our study were men (66.6%) and were over 40 years old.

On the other hand, we selected patients with HIV infection with more than 10 years of evolution, which may have influenced the high prevalence of atheromatosis observed in our patients.

### Factors related to the type of study

High variability is evident among published reports on the SAT prevalence in the HIV population. These diverse findings can be explained by the heterogeneity of the populations studied and the different criteria used to define atheromatous plaque. A total of 83.32% of the plaques found in our patients were in the carotid territory, and if we exclude the plaques found in the femoral territory, the prevalence of plaque was 52%, which was even higher than published rates. However, the number of territories studied must be considered. In most studies, the estimation of atheromatous plaque is performed on the common carotid [11], while in our case, we performed this estimation on the common carotid, bulbar, internal carotid, external carotid, common femoral and superficial femoral territories.

HIV infection is related to selective depletion of CD4 lymphocytes and increased expression of CD8, which lead to an inverted CD4/CD8 ratio (< 1) [13]. This ratio has recently emerged as a marker of immunosenescence and is independently associated with risk factors for both AIDS and non-AIDS events and mortality. In addition, it has been independently associated with intima-media thickness (IMT) progression [14] and SAT [15]. Specifically, a CD4/CD8 ratio < 0.5 or < 0.3 has been determined to be a risk factor for non-AIDS events or mortality, especially when the CD4 count is high (≥ 500 cells/μL) [16].

A decreased ratio can be maintained despite having achieved viral suppression and normalization of CD4 levels with ART [17]. Among the factors associated with a persistent inversion of the CD4/CD8 ratio are the presence of positive CMV serology, ART initiation before 1997, a low CD4 nadir and a short duration of viral suppression [18]. An inverted CD4/CD8 ratio and a low CD4 nadir have been associated with an increased risk of non-AIDS events [19].

The CD4/CD8 ratio tends to present greater stability over time than the absolute counts of CD4 and CD8 cells, and its predictive value for events unrelated to AIDS has been described as exceeding that of the absolute CD8 count in individuals with restored CD4 counts (> 500 cells/µL) [20].

This ratio can be used as a marker of immune dysregulation and as a predictor of non-AIDS events in these patients*.*

In our cohort, we observed an association between inversion of the CD4/CD8 ratio and the presence of carotid and femoral atheromatosis, which may constitute a parameter to be considered when estimating the risk of CVD in the HIV population without previous events. In addition, the presence of atheromatous plaque was associated with a low CD4 nadir and positive CMV serology, with no differences in viral suppression time found.

Through the use of predictors included in the logistic regression analysis, we obtained a diagnostic yield measured by the ROC curve of 72%.

In our cohort, we observed greater use of PIs in the SAT group. These results must be considered with caution, since patients with HIV follow different therapeutic regimens throughout their disease courses, and within the PI group, the new generations of PIs are very different in terms of side effects and cardiovascular safety.

Although studies related to normalization of the CD4/CD8 ratio with early ART initiation have been published [20–22], no differences have been found among the different classes of ART used [23].

Although the earliest PIs, such as ritonavir, have been associated with an increased risk of CVD [24], the use of new therapies, such as atazanavir, has been associated with slower progression of atheromatous plaque and a lower incidence of CVD [25].

Vascular ultrasound of the carotid and femoral territories allows assessment of the presence of atheromatous plaque in these locations and provides information on the extent of atherosclerosis at the systemic level.

This approach is fast and innocuous and may be useful to reclassify the vascular risk of people with HIV [26], allowing treatment readjustment with the objective of reducing the vascular risk and avoiding CVD in the future.

Performing ultrasound in primary prevention in intermediate-risk patients is relevant since it can permit vascular risk reclassification.

However, despite the high prevalence of plaque in the HIV population, this technique is normally not included in routine check-ups for several reasons, including a lack of ultrasound time and a necessary learning curve.

Identifying the predictive factors for the presence of SAT is essential to be able to reclassify patients and select those who will obtain a greater benefit from evaluations for SAT.

Therefore, the findings obtained in our cohort may be relevant to vascular risk assessments in these patients.

### Advantages

The CD4/CD8 ratio can play a role in comprehensive assessments of vascular risk in people with HIV, constituting a simple and accessible tool.

Through three clinical-analytical parameters easily extracted from the clinical history of participants (CD4/CD8 ratio less than 0.7, age and active smoking), we were able to predict the presence of SAT in HIV patients without previous CVD.

### Limitations

As the sample size was small due to the design of the study, these results should be considered with caution, and additional studies are necessary to confirm the findings.

A tendency towards a greater incidence of traditional vascular risk factors, such as hypertension and diabetes, was evident in the group with atheromatous plaque, although without significance, probably due to the insufficient sample size. We must also consider the greater use of PIs by patients with atheromatous plaque, which have been associated with an elevated vascular risk in people with HIV in other studies.

## Conclusions

With three simple parameters—tobacco, age and a CD4/CD8 ratio less than 0.7—we were able to predict the presence of atheromatous plaque in HIV participants without previous CVD with a good diagnostic yield (AUC: 0.72; 95% CI: 0.64–0.80). The CD4/CD8 ratio is a biomarker that may be relevant in comprehensive assessments of people with HIV. A CD4/CD8 ratio < 0.3 was associated with a 100% presence of SAT in our sample (95% CI: 81.6–100%).

## Data Availability

The datasets generated and/or analysed during the current study are not public but are available from the corresponding author upon reasonable request.

## Abbreviations

AIDS: Acquired immunodeficiency syndrome
ARTs: Antiretroviral therapies
AUC: Area under the curve
CVD: Cardiovascular disease
CVRFs: Cardiovascular risk factors
HCV: Hepatitis C virus
HDL-C: High-density lipoprotein cholesterol
HIV: Human immunodeficiency virus
INSTIs: Integrase strand transfer inhibitors
LDL-C: Low-density lipoprotein cholesterol
NNRTIs: Non-nucleoside reverse transcriptase inhibitors
NRTIs: Nucleoside and nucleotide reverse transcriptase inhibitors
PIs: Protease inhibitors
ROC: Receiver operating characteristics
SAT: Subclinical atheromatosis
TIA: Transient ischaemic attack
